# What is required in terms of mass drug administration to interrupt the transmission of schistosome parasites in regions of endemic infection?

**DOI:** 10.1186/s13071-015-1157-y

**Published:** 2015-10-22

**Authors:** RM Anderson, HC Turner, SH Farrell, Jie Yang, JE Truscott

**Affiliations:** London Centre for Neglected Tropical Disease Research, London, UK; Department of Infectious Disease Epidemiology, School of Public Health, Faculty of Medicine, St Mary’s Campus, Imperial College London, Norfolk Place, London, W2 1PG UK

## Abstract

**Background:**

Schistosomiasis is endemic in 54 countries, but has one of the lowest coverages by mass drug administration of all helminth diseases. However, with increasing drug availability through donation, the World Health Organisation has set a goal of increasing coverage to 75 % of at-risk children in endemic countries and elimination in some regions. In this paper, we assess the impact on schistosomiasis of the WHO goals in terms of control and elimination.

**Methods:**

We use an age-structured deterministic model of schistosome transmission in a human community and the effect of mass drug administration. The model is fitted to baseline data from a longitudinal re-infection study in Kenya and validated against the subsequent re-infection data. We examine the impact on host worm burden of the current treatment trend, extrapolated to meet the WHO goals, and its sensitivity to uncertainty in important parameters. We assess the feasibility of achieving elimination.

**Results:**

Model results show that the current treatment trend, extrapolated to the WHO goals, is able to greatly reduce host worm burdens. If coverage is continued at the same level beyond 2020, elimination is possible for low to moderate transmission settings, where transmission intensity is defined by the basic reproduction number, R_0_. Low levels of adult coverage have a significant impact on worm burden in all settings. Model validation against the re-infection survey demonstrates that the age-structured model is able to match post-treatment data well in terms of egg output, but that some details of re-infection among school children and young adults are not currently well represented.

**Conclusions:**

Our work suggests that the current WHO treatment goals should be successful in bringing about a major reduction in schistosome infection in treated communities. If continued over a 15 year period, they are likely to result in elimination, at least in areas with lower transmission.

**Electronic supplementary material:**

The online version of this article (doi:10.1186/s13071-015-1157-y) contains supplementary material, which is available to authorized users.

## Background

The third progress report of the London Declaration records that schistosomiasis remains red in the progress score card chart (recently developed by Uniting to Combat NTDs) as it has the lowest coverage of all helminth diseases treatable by mass drug administration (MDA) at 14.4 % in 2012 and 15.6 % in 2013 [1]. In addition, new mapping of schistosomiasis in affected countries is increasing the number of identified districts with endemic infection. Only twenty-six countries (50 %) of 52 endemic countries reported MDA in 2013 [1]. The report argues that significant improvements could be made in the coming years as drug supply of praziquantel is expected to increase and the launching of the new Global Schistosomiasis Alliance will increase collaboration within this disease community to help countries scale up MDA of children and adults [2].

Global treatment numbers for schistosomiasis have slightly improved in recent years. In 2013, 47.3 million people were treated with praziquantel, while the comparative figure was 42 million in 2012. However, donations of praziquantel are increasing significantly. In 2014, pharmaceutical donor Merck increased its contribution to nearly 75 million tablets, and of 41 African countries requiring treatment, 36 (88 %) were reached. Merck has increased its donation in 2015 to 100 million tablets. For 2016, Merck has committed to increasing its donation of praziquantel up to 250 million tablets, equivalent to 100 million treatments. As reported in the third progress report, DFID, USAID, the World Bank, and World Vision also purchased over 100 million of praziquantel tablets in 2015 [1].

**Fig. 1:**
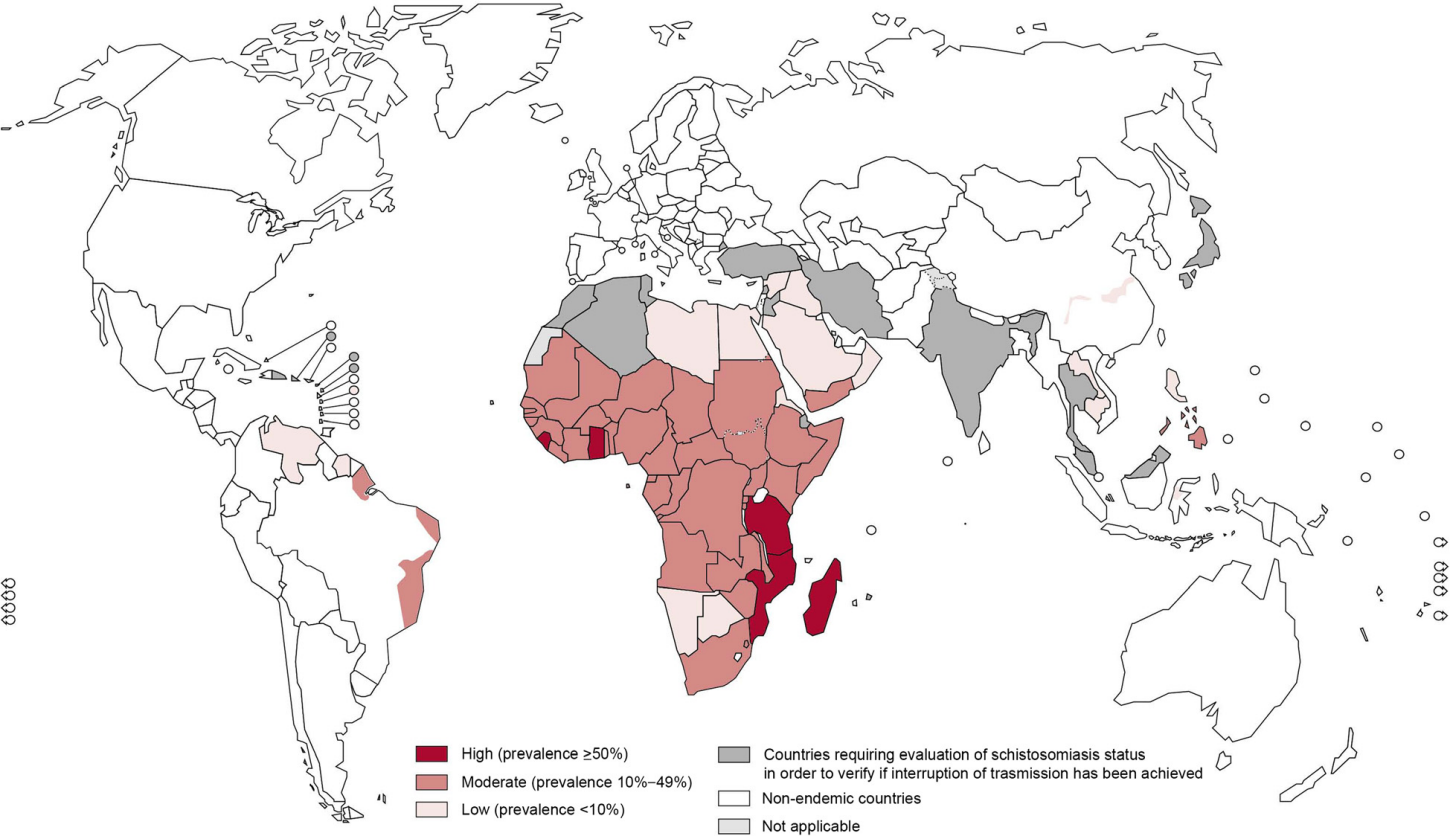
Distribution of schistosomiasis worldwide in 2013 as reported by the WHO [1]

The targets set by the World Health Organization (WHO) at the 2015 World Health Assembly (WHA) for schistosomiasis control are elimination as a public health problem, by 2015 in the Eastern Mediterranean, Caribbean. Indonesia and the Mekong River Basin, and by 2020 regionally in the Americas and Western Pacific and nationally in selected African countries (Fig. 1). To achieve these ambitious targets WHO aims that 75 % of school-aged children (SAC) in need of preventive treatment will be regularly treated in 100 % of endemic countries by 2020 [3]. For these targets elimination is not defined clearly, nor is the frequency of treatment required.

In this paper, we address what might be required to eliminate transmission for *Schistosoma mansoni* using annual MDA coverage in pre-SAC, SAC and adults. We also assess the impact on host worm burden of historic coverage levels, projected forward to 75 % in SAC by 2020. Only in six of 31 reporting countries was the target of reaching at least 75 % of school children achieved [4].

To provide quantitative predictions of MDA impact we employ deterministic age structured mathematical models that incorporating sexual reproduction, aggregated distributions of worm numbers per host (described by the negative binomial distribution with aggregation parameter k), density dependent egg production and variable contact with, and production of, infective stages to continue the life cycle, by different age groupings of the host population, defined as pre-school aged children (pre-SAC, 0–4 years of age), school aged children (SAC, 5 to 14 years of age), and adults (> 15 years of age). The model template is described in Anderson & May [5] and modifications to this structure to include variable exposure to infection and production of eggs by host age groupings are detailed in Truscott et al. [6].

## Methods

Details of the mathematical model employed are described in previous publications [6–8] and also in Additional file 1. Most research on model development for schistosome transmission is based on the pioneering studies of Macdonald in the 1960s [9]. He was the first to point out that sexual reproduction in dioecious species created a breakpoint in transmission (an unstable equilibrium separating the two stable points of endemic infection and parasite extinction) below which mating frequency is insufficient to sustain transmission. More recent modifications include the inclusion of negative binomial distributions of parasite number per host [10, 11]. Worm counts based on autopsy studies show clearly that parasites are highly aggregated within the host population where most hosts harbour few parasites and a few harbour many [12]. These autopsy results are also confirmed by plots of the prevalence of infection versus mean epg counts which also show high degrees of aggregation [13, 14].

Other important modifications in past work include the demography of the human host population [15], the impact of MDA [16–18], acquired immunity (dependent on past exposure to infection) [10], spatial structure and host gender [19], density dependence in egg production [20], predisposition to heavy infection [13], stochastic individual based models [20] and the generation of parasite aggregation through heterogeneity in exposure amongst the host population [20, 21].

In this paper we employ a deterministic hybrid model which assumes the parasite is dioecious and monogamous, has density dependent egg production [20] and a degree of parasite aggregation defined by the negative binomial probability distribution with a fixed value of the aggregation parameter *k*. The details of the model can be found in the supplementary information. The within-host section of the model describes the evolution of the worm burden in individuals as a function of age. It has full age structure, but outputs are grouped into three age groupings for pre-SAC (0–4 years of age), SAC (5–14 years of age) and adults (>15 years of age), reflecting the key age distinctions made within MDA programs. We include variability in host exposure to infection and contributions to egg production dependent on host age group (described by a parameter *β*_*i*_ which describes the intensity of infectious contact of age group *i* with the infectious material in the environment. Because of the relative fast time scales of turnover in the larval stages and snail intermediate host of schistosomes (see review by Anderson & May [16]), relative to the long life expectancy of the adult worm in the human host, the details of the dynamics of the free living and snail based stages of the parasite are collapsed into an equation for infectious material in the environment.

**Fig. 2:**
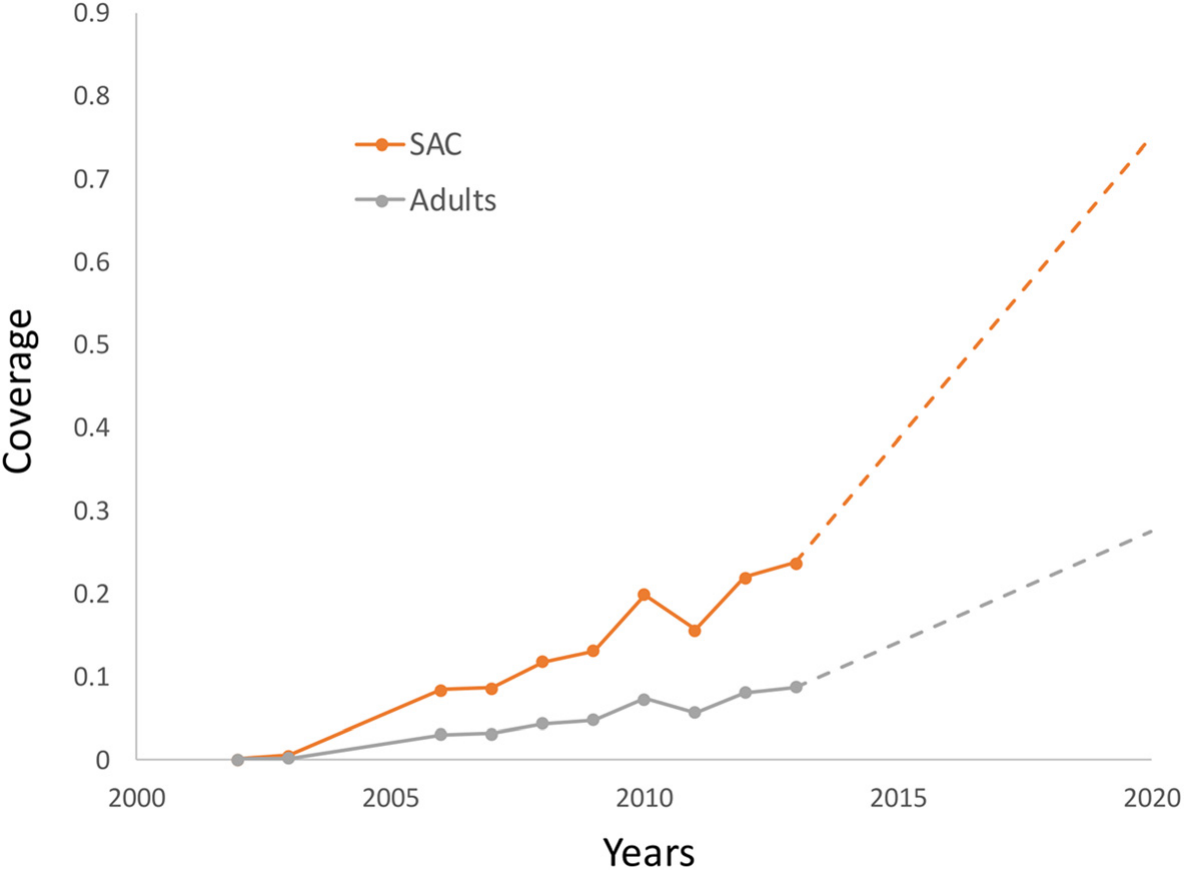
National coverage in endemic regions as reported by the WHO. The age-specific (SAC and adults) coverages, were estimated from the national figures by assuming that SAC account for 70 % of all those treated [4], and are 46 % of the current Population requiring PC (based on PCT data bank [22]). The trends in SAC and adult coverage are represented by the orange and grey lines, respectively. Solid lines represent reported figures and broken lines the project trends in coverage to reach 75 % coverage for SAC in 2020 and maintain the current ratio of SAC/adult coverage. In model projections, coverage levels are assumed to be constant after 2020

The history of treatment coverage with praziquantel in the SAC age group (5–14 years of age) and adults combined as reported by WHO is as illustrated in Fig. 2 [4, 22]. We have partition the national coverage data to give values for SAC and adults separately. Coverage of SAC for the years after 2014 was constructed as a linear trend from the last data time-point to an assumed level of 75 % in 2020 and beyond. Adult coverage was constructed to remain in constant proportion to SAC coverage over the period 2014–20, reaching a plateau at about 27 %. In a recent review the mean egg reduction rate (ERR) reported for a 40 mg dose of praziquantel was 95 % for *S. japonicum*, 94.1 % for *S. haematobium*, and 86.3 % for *S. mansoni*. The drug currently is not recommended for use in pre-SAC [23].

**Fig. 3:**
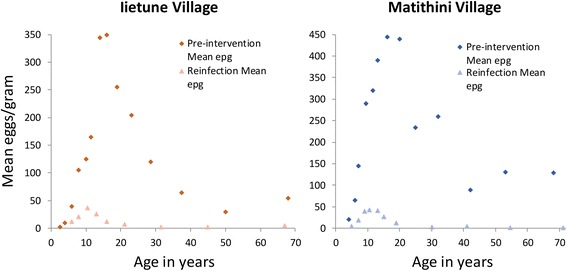
Age mean intensity (epg) profiles for *S.mansoni* in two Kenyan villages from Fulford et al. (1995) pre (solid line) and post (dashed line) MDA with praziquantel. Note the large impact that MDA has in these settings. The village in Graph B was used in parameter estimation employing likelihood methods to fit the model to the observed mean epg counts

**Fig. 4:**
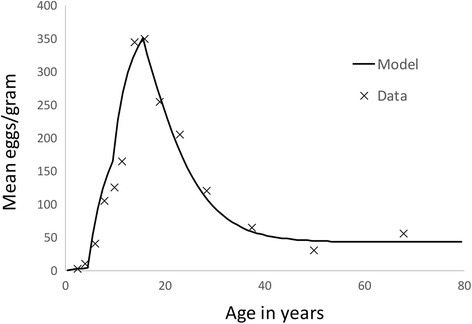
Maximum likelihood fit of the deterministic model to age cross sectional data of the mean intensity of infection (epg counts) from Iietune village in Kenya (Fulford et al., 1995) as plotted in Fig. 3. The solid line is the best fit and the crosses are the raw data. The estimated parameter values are recorded in Table 1

Parameter assignments for the major population and transmission processes were as defined in Table 1 based on the extraction of values from epidemiological studies of *S mansoni* in Kenya by Butterworth and colleagues using a full cross sectional age - average intensity profile [24–26]. Parameters were estimated using maximum likelihood techniques applied to the mean epg age profiles. Parameters not well informed by this data were taken from other studies in the literature as recorded in Table 1. The age intensity profiles (based on mean epg counts) pre- and post-treatment are recorded in Fig. 3. A fit of the model to one set of data is recorded in Fig. 4.

**Table 1:** Parameter values used in the numerical evaluations of model predictions for *S mansoni*. The age intensity profile from the cited sources based on faecal egg counts were used to derive estimate of R_0_ and β_i_ using maximum likelihood methods. The age groupings for the contact parameter β_i_ are defined as the intervals between 0, 5, 10, 16 and 80 years

Parameter	Value	Source
Basic Reproductive number, R_0_	2.00	Fitted
Adult worm life expectancy	4 years	[10]
Negative binomial clumping parameter, k	0.24	[34]
Density dependence fecundityparameter, ɣ	0.0006/female worm	[34]
Beta, β_i_ for intensity of age group i’s contact with infectious material	0.22,1.88,1,0.53	Fitted
Drug efficacy as a proportion of worms killed by praziquantel	0.86	[44]
Egg output per female worm	0.14	[45]
Source of age - mean intensity data	-	Ietune village, Machakos District, Kenya [26]

The issue of whether or not the convex age intensity curves recorded for the schistosomes are due to acquired immunity or age related exposure remains uncertain. The problem of observed epidemiological pattern interpretation was first enunciated by Warren in 1973 who described the problem succinctly as one of differentiating between ecology and immunology [27]. The conundrum in interpretation remains today, although there is abundant clinical evidence of the mounting of strong and specific immunological responses to schistosome infections by the human host [28]. In our analyses of the impact of MDA, we assume that the convex age intensity profiles are generated by age dependent exposure. Future work will assess the impact of weakening this assumption to include the more realistic premise that both ecology and immunology act as determinants.

Treatment coverage is assumed to be at random at each round of MDA in each age group. This is unlikely to be true in reality and a future publication will examine adherence issues within a stochastic framework. If those not treated are predisposed to this state for social or behavioural factors, the impact of MDA at 75 % coverage will be less than predicted in the results discussed in this paper.

Host demographic details (age dependent death rates and population pyramids) were taken from published data on sub-Saharan countries with the profile in Uganda chosen to represent a typical profile for sub-Saharan Africa where *S. mansoni* is endemic [29].

### Model validation

Validation of model predictions is essential and can be performed in variety of ways. Qualitative comparisons of model prediction with observed pattern of reinfection post mass treatment is one approach. Ideally, however, quantitative comparisons of predicted outcome of the impact of MDA require parameter estimation using data from a defined population with little or no recent past treatment and then the comparison of expected and observed patterns of reinfection at subsequent rounds. Given the age structured nature of the current model, a detailed validation requires precise information on who has been treated, at what age, how often and their representation within the final reinfection survey. For schistosomes , worm loads are usually measured indirectly by eggs per gram of faeces (epg) or per unit volume of urine in the case of *S haematobium*. This measure is known to be unreliable, typically underestimating the true prevalence. Furthermore, in estimating intensity, it has a high variance as recorded in repeated samples from the same stool and in stools taken on consecutive days [30]. New tools, such as quantitative PCR, offer some hope for the future in providing precise scores for prevalence, and perhaps even egg burdens in stools. A further complication arises from density dependence in egg production by female worms which can result in higher per capita egg output post the initial round of treatment during the early phases of reinfection.

Model validation has rarely been attempted for helminth infections, but it is a central need for ongoing and future epidemiological studies of MDA impact, if predictions on coverage requirements to interrupt transmission are to carry weight amongst public health policy makers. A previous attempt by French et al. (2010) used pre and post treatment epg counts from a study of *S mansoni* in Uganda recording reinfection over 3 years of follow up, but validation was based on estimating parameter values from the trends in reinfection [17]. Ideally, it should be done by estimating these parameters first, and then predicting reinfection pattern to compare with observed outcomes given data on treatment coverage.

We report a validation exercise using a reinfection study of *S mansoni* recording prevalence and mean epgs in a set of villages in Kenya that had not previously been treated prior to the initiation of mass drug administration [26]. We also examine the sensitivity of model predictions of the impact of achieving 75 % coverage in SAC to changes of R_0_, to different values of the contact parameter β_i_ and to the level of drug coverage especially in adults.

## Results

**Fig. 5:**
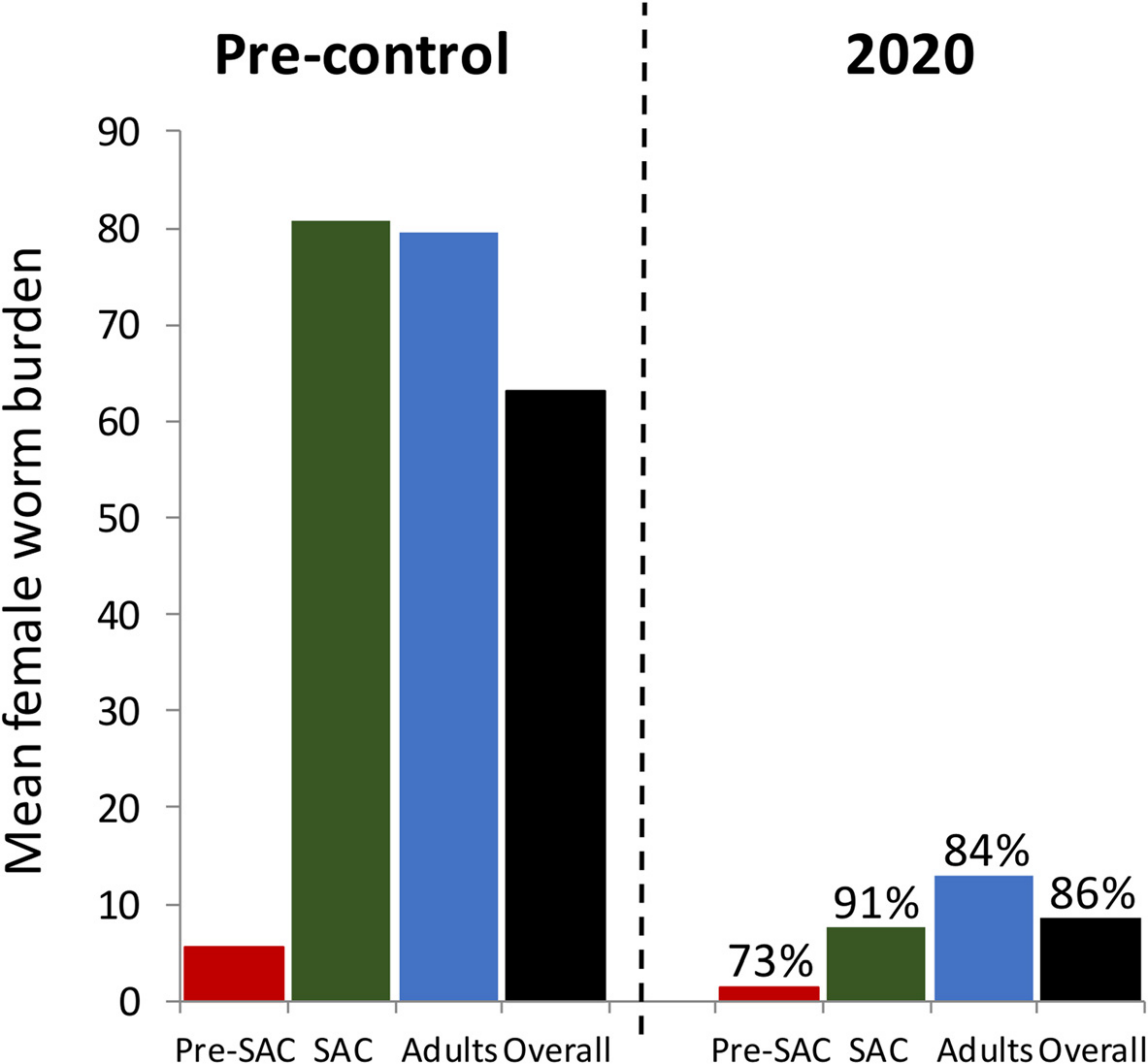
Changes in the mean worm burden in the three age groups and in the overall population mean between 2001 and 2020 with coverage as defined in Fig. 2. Percentages indicate the worm burden reductions in the respective age groups

We present the results of the numerical evaluations of model behaviour under the coverage trends shown in Fig. 2 and the parameters described in Table 1. Figure 5 displays a histogram recording the initial mean female worm burden in 2001 in pre-SAC (<5 years of age - no treatment coverage), SAC (5–15) and adults (15+ years old), and the respective worm burdens at year 2020 resulting from the treatment coverage trend shown in Fig. 2. Note that the clear peaked profile shown in Fig. 4 is lost when averaging across pre-SAC, SAC and adult age categories as a result of the positioning of the peak at the boundary of the SAC and adult classes. It’s clear that achieving 75 % coverage in SAC and 27.4 % in adults via annual rounds of treatment has a very considerable impact on the mean intensity of infection in all age groups. The gradual achievement of this impact can be compared to the rapid achievement of a similar level of control after 2 rounds of treatment achieved by Butterworth and colleagues in their original study, as recorded in Fig. 3 [26]. In the latter case, levels of coverage in excess of 90 % were required across the entire age range of the population (with the exception of those under 5 years).

**Fig. 6:**
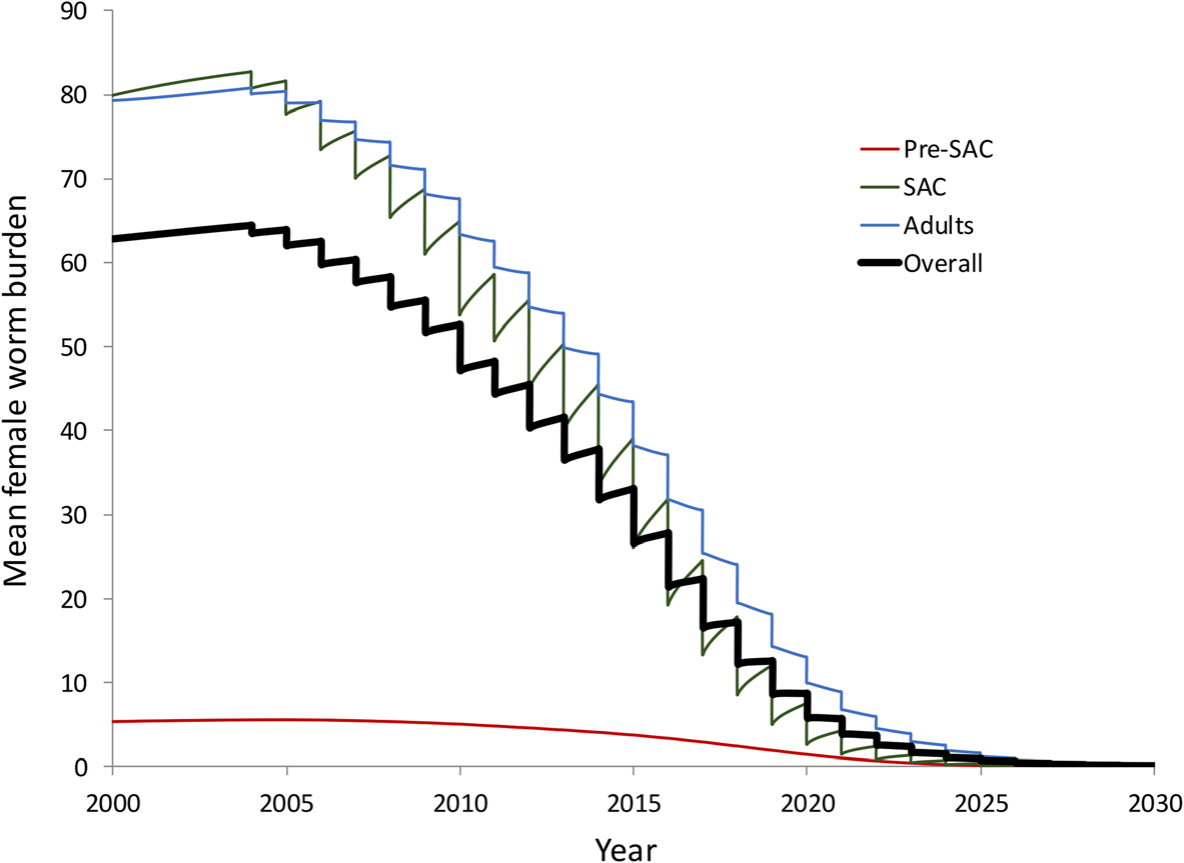
Predicted time series for mean intensities of infection (epg) in pre-SAC, SAC, adults and overall from 2003 to 2020, with the coverage trends defined in Fig. 2. After 2020 the coverage is assumed to remain constant at 75 % in Sac and 27.4 % in adults

Figure 6 plots the time series underlying these calculations (with parameter values as in Tables 1 and 2), with treatment coverage extended beyond 2020 at the levels achieved in 2020. Note that at a level of 75 % coverage in SAC and 27.4 % coverage in adults the model predicts that the transmission breakpoint is crossed and transmission is extinguished by 2030.

**Table 2:** Sensitivity analyses of model projections to changes in the values of key parameters. The numerical values record the percentage reduction in the predicted mean intensity of infection in each age group from a rising treatment coverage to 75 %, and to 27.4 % in adults, in 2020 as compared to baseline in 2003

Parameter	Pre-SAC mean burden	SAC mean burden	Adults mean burden	Overall population mean burden
Fitted parameter values (Table 1)	73 %	91 %	84 %	86 %
Higher and lower transmission setting (R_0_ + 1.0, −0.25)	49 %, 81 %	78 %, 94 %	76 %, 86 %	76 %, 90 %
Coverage level achieved in 2020 (60 %, 90 %)	77 %, 67 %	94 %, 85 %	87 %, 79 %	90 %, 81 %
Degree of exposure and contribution of children to the infectious reservoir (5–16 years) (β_2_ and β_2_ + 50 %, −50 %)	73 %, 71 %	91 %, 90 %	84 %, 82 %	87 %, 85 %

### Sensitivity analyses

We performed a basic sensitivity analysis on the impact of the treatment coverage trend with respect to some of the more important or uncertain parameters, the results of which are summarised in Table 2. Transmission intensity, as captured by R_0_ in our model, is encapsulates a large number of features of the transmission cycle and is likely to vary from community to community. The value obtained from the current fit (R_0_ = 1.7) is relatively low with respect to previous estimates [5, 31]. An increase of R_0_ by 1 decreases the impact of treatment by 2020 considerably. However, examination of the time series suggests that elimination is still possible, but delayed beyond 2030.

The coverage trend shown in Fig. 2 is an average across many countries in which schistosomiasis is endemic. Across individual communities, however, levels of coverage are likely to vary considerably. Table 2 shows that the impact of the 2020 level of coverage of children is relatively small and also linear across all age groups. This indicates that, even though coverage may vary across treated communities in a country, average coverage levels will give a good estimate of the average effect on worm burden in the population as a whole.

Coverage of adults is not currently part of the WHO goals for schistosomiasis, but treatment across the whole community has also been shown to be highly effective in the Machakos study [26]. Table 3 shows that treatment of adults has a relatively small impact on worm burden reduction overall, with the effect diminishing as coverage gets higher. This insensitivity reflects the low schistosome burden in adults in the data to which we fitted the model. For other datasets (such as that for Matithini village in the same study), a higher burden in adults may translate to greater sensitivity to adult coverage levels.

**Table 3:** Sensitivity of model projections (percentage reduction in the predicted mean intensity of infection) to changes in the level of coverage of adults achieved in 2020 (linear trend from last data point in 2013)

Adult coverage by 2020	Pre-SAC mean burden	SAC mean burden	Adults mean burden	Overall population mean burden
15 %	72	90	78	83
30 %	73	91	85	87
45 %	74	92	89	90
60 %	75	92	93	92
75 %	76	93	95	94

### Interrupting transmission

**Fig. 7:**
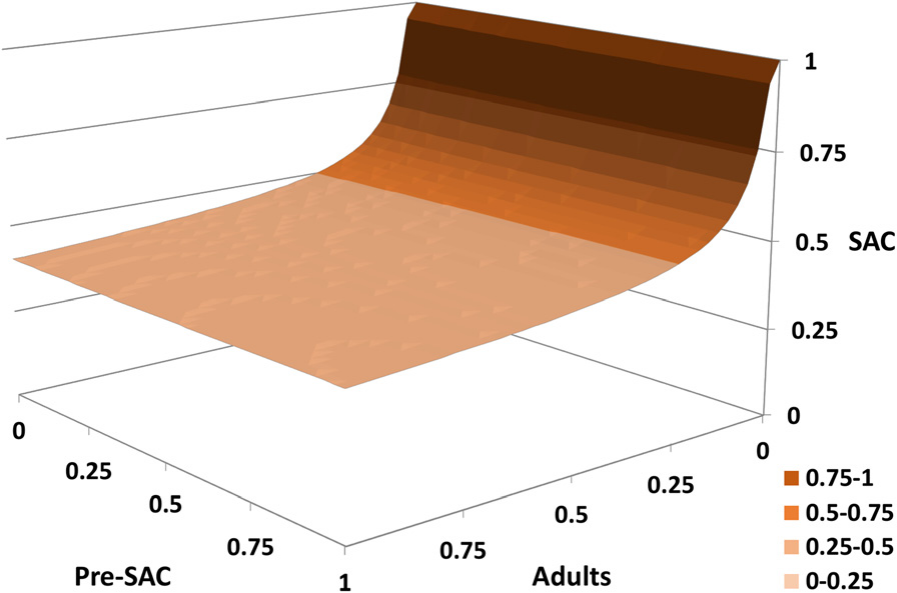
‘Elimination surface’ for *S mansoni* calculated for the parameter assignments listed in Table 1. Levels of coverage above the surface are predicted to reduce worm burden in the population within 15 years to such a level that it cannot subsequently recover

The current ambitions of WHO include the aim of interrupting transmission in defined settings with 75 % coverage of SAC and some undefined coverage level in adults [3]. We examine the feasibility of achieving this aim with various levels of treatment coverage in pre-SAC, SAC and adults by plotting an ‘elimination surface’ for treatment coverage required to interrupt transmission. This is shown in Fig. 7 which records the values of coverage in pre-SAC, SAC and adults above which, if continued for 15 years, reduce worm burden to such a level that it cannot subsequently recover. These ‘breakpoint surfaces’ have been reported in other publications for soil transmitted helminths [6, 7, 32]. This is the first time they have been calculated for schistosomes. Note that the predictions are generated under the assumption of monogamy amongst adult worms. If a degree of polygamy pertains, the surface will be higher on the SAC axis since the breakpoint will be more difficult to reach [5, 11].

Much of the shape of the elimination surface can be understood with reference to the baseline age profile shown in Fig. 4. For example, egg output from the pre-SAC age group is -quite low and this corresponds with the low sensitivity of elimination to coverage in pre-SAC (the surface is very flat in the direction of changing pre-SAC coverage). Treatment of pre-SAC is not currently part of the WHO goals for schistosomiasis. The drug is not authorised for use in this age group (see Methods section), although efforts are being made to develop a suitable formulation [33]. However, our results suggest that they would be of limited use in bringing about elimination, although they could be important in reducing morbidity. Coverage of adults is important at low levels, enabling elimination to be achieved within the WHO goals for SAC. Further increase in adult coverage has little additional effect due to the relatively low worm burdens among adults. The impact of treating adults is reduced if a longer period of treatment is recovered, showing that the treating adults reduces the time to elimination rather than the long-term possibility of elimination.

The close association between the baseline age-profile and the elimination surface suggests that if the baseline varies between populations, the conditions under which elimination can be achieved will vary too. For example, other age profiles have a stronger presence in worms within the adult population and this will translate into a greater sensitivity to adult coverage.

The predictions reported in this paper are for *S mansoni*, but given that the life expectancy of *S heamatobium* and its age-profile within the host population are similar, the predictions should apply for that species too. In the case of *S. japonicum*, the added complexity of multiple zoonotic reservoirs will likely mean that conclusions drawn from *S. mansoni* cannot be directly transferred to the other species that infest humans.

### Model validation

As outlined in the Methods section, validation of model predictions requires comparison of observed outcomes with corresponding post-treatment predictions, given baseline initial conditions and parameter estimates and data on who is treated, how frequently and who is surveyed. Given the nature of our model, ideal data would be individual longitudinal egg count data with matching data on age and treatment history.

Few published epidemiological studies on schistosomes have this level of detail, at least in their published form. The study by Butterworth et al. of *S mansoni* transmission and treatment in Machakos district, Kenya offers a good compromise in that it reports age group-stratified baseline and re-infection data across several study arms with data on MDA coverage and timing over a number of years [24, 26, 34]. We focus on the study arm in Iietune village. In this population, an initial survey in 1983 was followed immediately by a round of MDA. A second round occurred in 1985 and a re-infection survey was completed in 1987. A coverage of 93 % was achieved in both rounds, assumed for the purpose of validation to be coverage of those eligible for treatment (5 years or over). We have extracted epg data from the baseline and the re-infection survey, with the baseline data being used to parameterize the model using maximum likelihood techniques.

**Fig. 8:**
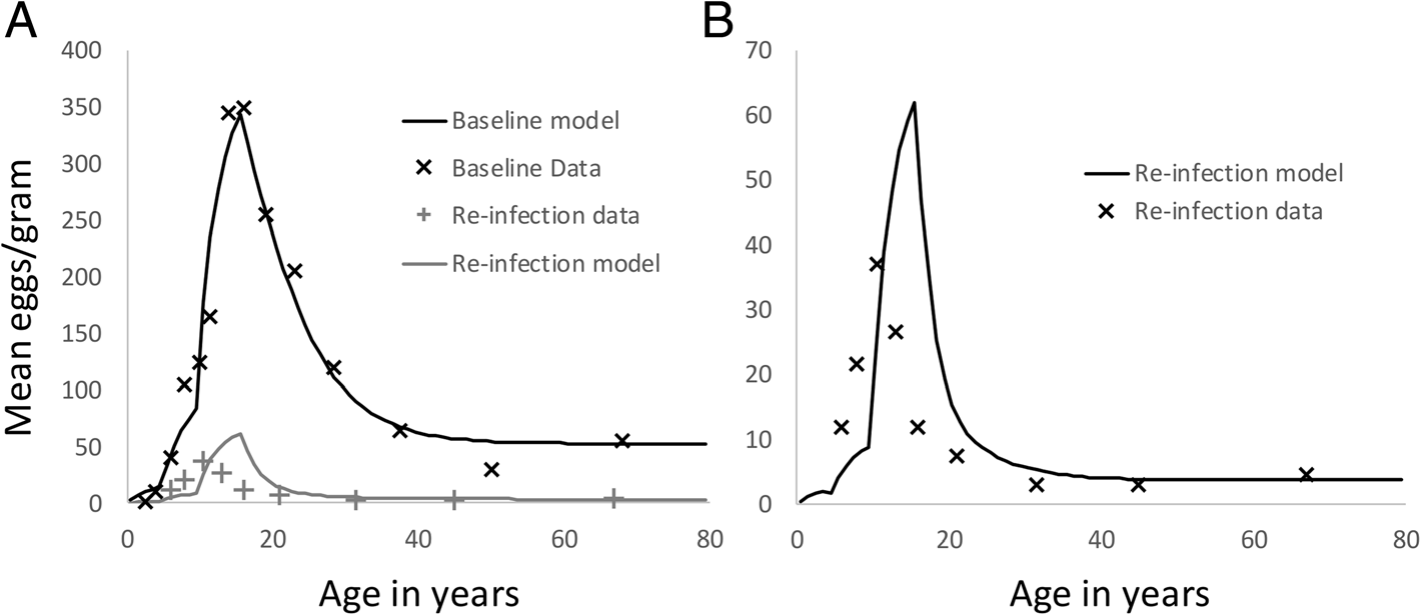
Validation of model behaviour against baseline and reinfection data from Iietune village, Machakos district, Kenya. Panel A shows the fitted equilibrium age profile from the model against baseline data from 1983 and the response of the model in 1987 after two rounds of treatment (1983, 1985). Panel B shows the details of the model age profile against the re-infection survey data

The results of the validation analysis on the Fulford et al. data is displayed in Fig. 8. Panel A records the fit of the model to the observed mean egg counts for *S mansoni* in each recorded age group at baseline and also the comparison between the re-infection data and the model’s prediction. Note that no revised parameter estimates where made using the reinfection data – so this is ‘true’ validation, in the sense of using the predefined model to make predictions of MDA impact. It’s clear that the correspondence with the re-infection data is approximately correct on average, but there are some discrepancies in the distribution of parasite burden among school-age children and young adults. Panel B shows the details of predicted and observed re-infection profile. The model has successfully captured re-infection levels among older adults (20+ years), but has overestimated the levels among individuals in the 10–20 year age bracket. One possible reason is that the assumption of a single infectious reservoir is incorrect. It is possible that young children have a tendency to form a ‘core re-infection group’. Without this mechanism, re-infection in this group will be under-represented by the model relative to the older children, shifting the infection peak towards older ages. Another potential reason is immune response to infection and treatment which is not currently captured by the model. These issues will be explored in future work.

## Discussion

The general conclusion generated by the model predictions is that an annual 75 % coverage in SAC and a modest level of coverage in adults should reduce mean intensities of infection to very low levels by 2020. In low to moderate transmission settings continuation of this coverage for a decade or so should result in transmission elimination. For high transmission settings, however, somewhat higher levels of coverage will be required. These predictions seem to be relatively insensitive to changes in the parameter estimates explored in Table 2.

The key question, however, is whether coverage can be raised to 75 % in SAC and modest levels in adults? Concomitantly, can drug production levels also be raised to meet such demands? These questions are beyond the scope of this paper and the resolution of them lies with WHO, policy makers, implementers within countries and the pharmaceutical companies who makes the major donations of praziquantel.

Model validation against reinfection data indicate that, while broadly in agreement with the data, there are clear discrepancies. These arise from two equally important sources. On the one hand, the failure to capture the behaviour of the population in the 12–25 year age range suggests that a more sophisticated model of infectious contact among children is required. On the other, there is a lot of missing and uncertain material in the data being used in this validation, such as past history of treatment (which we have assumed no past history). The availability of high quality data is a major issue for model testing and validation. Far too few published studies record data on intensity and prevalence by age group (ideally individual person epg counts) electronically and make such data freely available to other researchers. This is an important need in the coming years as MDA coverage is hopefully increased.

As it currently stands, our model does not include the effects of immunity. Immune responses are not well understood, but possible mechanisms include acquired immunity from past exposure to infection and a long-lived immune response generated by death of adult worms [35, 36]. Without a clear understanding of the mechanisms of acquired immunity, it is very hard to disentangle the effects of immunity from age-dependent exposure to infection (β). However, re-infection data, stratified by age, may offer a chance to distinguish the two processes. Our current model overestimates reinfection in an age-group that was heavily infected at baseline, perhaps suggesting that an immune response triggered by treatment may be responsible. Future work will examine this possibility.

Another potential source of error in our model is the treatment of host demography. The data used for both fitting and validation is from studies conducted in the 1980s, but our modelling concerns the past decade and near future. Within this interval, significant changes in demography have certainly taken place. We use a frequency-dependent model of infection whose behaviour will not be effected by the absolute host population (whether absolute population size effects disease dynamics in reality will require detailed analysis of datasets from different time periods). More significant are changes in the age distribution of the host population which will affect the mean force of infection experienced by individuals and their contributions to the infectious reservoir. Such effects can be investigated with our model, but will require high quality infection data from a range of different demographies.

Broader scientific issues relate to the uncertainties on biological questions, the quality of data available for parameter estimation, data on drug coverage levels and the impact of heterogeneities in transmission. Biological uncertainties are key in two areas. The first is the question of monogamy for adult parasites. The calculations recorded in this paper will be extended in future work to look how the assumption of polygamy influences predictions. In rough terms it will make elimination of transmission a little more difficult (raising the surface in Fig. 7).

The second biological question relates to the importance of acquired immunity. Again, this requires further exploration. Previous studies have outline various model frameworks for helminths to address this issue using infection functions that depend on past exposure to infection and these will be employed in future analyses. However, it is possible to speculate on what influence acquired immunity may have [35]. Broadly speaking, if immunity is important and builds up through early life exposure, then repeated MDA will act to reduce herd immunity in a manner that will increase reinfection rates after many rounds of treatment as children lack past experience of infection. This would act to lessen the long term impact of MDA and require the elimination of transmission to make sure that, if treatment stopped (or decreased), the population was not more susceptible to infection and associated morbidity.

Data availability to test predictions against observations is good in one context and poor in others. Epidemiological data, well stratified by age and sex, on reinfection post rounds of MDA is good, as well illustrated in Fig. 3 by the studies of Butterworth and colleagues [17, 26]. Such data facilitates model validation.

Data is poor for the estimation of key population dynamic parameters such as density dependent fecundity and the relationship between epg measures and worm counts because direct measurements of host worm populations *in vivo* is impossible. Estimates can be obtained from experimental animal models (mice and some primate species [37, 38]) as well as from autopsy studies, although these are very rare and probably constitute a biased sample [39]. The alternative source for estimates for these parameters is through fitting complex models to extensive datasets [34]. In this sense, model development and parameterisation for schistosome species is much more difficult than for soil transmitted helminths, where many worm expulsion studies have been published [40–42].

Drug coverage data for praziquantel treatment stratified by the age group treated is poor at present. This could be easily remedied by requesting countries to record this information as treatment coverage expands. This data is essential for making accurate predictions, given that for all schistosome species, a significant fraction of the total adult parasite population is harboured by those over 15 years of age [43].

The transmission of schistosome species relies on human contact with water, and as such much heterogeneity exists between endemic settings in the exposure of individuals by age and gender as a consequence of the prevailing cultural, social and behavioural conditions. Our predictions were based on parameter values derived from one medium intensity transmission setting, but in the villages studied by Fulford et al. in Kenya much variation in exposure to infection was apparent village by village, relating to the type of water contact and what sort of aquatic habitat was present near each village [26]. Addressing such heterogeneity requires the use of individual based stochastic models incorporating spatial transmission, and these will be a priority in future research.

A stochastic framework will also facilitate addressing other important sources of heterogeneity, such as predisposition to heavy infection [13], and variability in, or predisposition to, treatment at each round of MDA. Data requirements in this context include the keeping of accurate records of precisely who receives treatment at each round of MDA.

## Conclusion

In conclusion, we return to the main prediction of the model calculations. *S. mansoni* control, and even the interruption of transmission, is possible if high levels of annual treatment coverage in SAC are reached and maintained, plus some modest coverage in adults of around 30 %. Reaching the 75 % coverage in SAC in all endemic regions is the challenge for the coming years.

## Additional file

Additional file 1:
**Supplementary information.** (PDF 445 kb)
