# Supplementary material for: What is required in terms of mass drug administration to interrupt the transmission of schistosome parasites in regions of endemic infection?
Source: Parasit Vectors. 2015 Oct 22;8:553. doi: 10.1186/s13071-015-1157-y (PMC4618750; doi:10.1186/s13071-015-1157-y)
Supplement: Additional file 1: — Supplementary information. (PDF 445 kb) [file 13071_2015_1157_MOESM1_ESM.pdf]

Supplementary information

The Model

The fundamental model used to describe the mean worm burden of individuals of a given age and the quantity of infectious eggs in the environment was developed from the founding work of Anderson and May [1]. The current version of the model is described in detail in [2, 3]. Briefly, the model is an PDE model describing the evolution of mean female worm burden as a function of age, *M*(a,t)

$$\frac{\partial M(a,t)}{\partial t}+\frac{\partial M(a,t)}{\partial a}=L\beta(a)-\sigma M(a,t)$$

where *L* is the concentration of infectious material in the environment. The model describes the evolution of the female worm burden and assumes they are distributed according to an underlying negative binomial distribution with fixed negative binomial aggregation parameter, *k*. The dynamics of infectious material is governed by

$$\frac{dL}{dt}=\psi\lambda\int_{a=0}^{\infty}M(a)f(M(a),z,k)\rho(a)P(a)da-\mu_2 L$$

where *P(a)* is the normalised age distribution for the population. The function $f(.)$ describes the production of fertile infectious material and is the product of a term representing the dampening effect of density dependent fecundity at higher worm burdens [first term] and the catalytic effect of the presence of male worms on sexual reproduction at very low worm burdens, ϕ

$$f(M,z,k)=[1+(1-z)M/k]^{-(k+1)}\phi(M,k)$$

where $z=e^{-\gamma}$ representing the strength of density-dependent fecundity and the parameter ψ characterises the flow of infectious material into the environment. Several previous models have included a dependence on worm burden in *k* [4, 5]. In the context of the response to the model to changing coverage levels, we found negligible sensitivity to the value of the aggregation parameter. However, we have not investigated its impact on elimination behaviour. Sexual reproduction is assumed to monogamous, dependant on male-female worm pairs. In this case, the appropriate form for the function ϕ is [6]

$$\phi(M,k)=1-\frac{1}{2\pi}\left(\frac{k}{M+k}\right)^{1+k}\int_{0}^{2\pi}\frac{(1-\cos(\theta))\,\mathrm{d}\theta}{(1-M\cos(\theta)/(M+k))^{(1+k)}}$$

The parameter ψ and the absolute magnitude of β and ρ are subsumed into the definition of the basic reproduction number, R_0, that measures the intensity of the transmission cycle.

$$R_0 = \frac{z\lambda\psi}{\mu_2} \int_{a=0}^{\infty} \rho(a)P(a) \int_{x=0}^{a} \beta(x)e^{-\sigma(a-x)}dxda$$

Assuming a 1:1 sex ratio in worms, the total worm burden is given by $2M(a,t)$.

Treatment is modelled without systematic non-compliance. Coverage in an age group is taken as the probability that an individual in that age group will receive treatment. Hence the drop in mean worm burden from treatment is the product of the coverage and drug efficacy (which can be understood as the probability of a single treatment killing a worm within the host).

The data to which the model was fitted were taken from the Machakos study by Butterworth and colleagues [7] and were available in the form of mean eggs per gram across an age group. Model output in the form of mean eggs per gram at a given age and time is given by

$$\bar{E}(a,t) = \lambda M(a,t) f(M(a,t), z, k)$$

where λ is the mean egg per gram output from a single worm pair in the absence of density-dependent fecundity effects (See Table 1).

References

1. Anderson RM, May RM: **Infectious diseases of humans : dynamics and control**. Oxford ; New York: Oxford University Press; 1991.
2. Truscott JE, Hollingsworth TD, Brooker SJ, Anderson RM: **Can chemotherapy alone eliminate the transmission of soil transmitted helminths?** *Parasites & vectors* 2014, **7**(1):266.
3. Truscott JE, Turner HC, Farrell SH, Anderson RM: **Soil Transmitted Helminths: mathematical models of transmission, the impact of mass drug administration and transmission elimination criteria**. In: *Mathematical Models for Neglected Tropical Diseases: Essential Tools for Control and Elimination. Volume B*, edn. Edited by Anderson RM, Basanez MG; 2015.
4. Chan MS, Guyatt HL, Bundy DA, Booth M, Fulford AJ, Medley GF: **The development of an age structured model for schistosomiasis transmission dynamics and control and its validation for Schistosoma mansoni**. *Epidemiol Infect* 1995, **115**(2):325-344.
5. French MD, Churcher TS, Gambhir M, Fenwick A, Webster JP, Kabatereine NB, Basanez MG: **Observed reductions in Schistosoma mansoni transmission from large-scale administration of praziquantel in Uganda: a mathematical modelling study**. *PLoS neglected tropical diseases* 2010, **4**(11):e897.
6. May RM: **Togetherness among Schistosomes: its effects on the dynamics of the infection**. *Mathematical biosciences* 1977, **35**:301-343.
7. Fulford AJ, Butterworth AE, Sturrock RF, Ouma JH: **On the use of age-intensity data to detect immunity to parasitic infections, with special reference to Schistosoma mansoni in Kenya**. *Parasitology* 1992, **105 (Pt 2)**:219-227.
